# Supplementary material for: Doubly robust augmented weighting estimators for the analysis of externally controlled single-arm trials and unanchored indirect treatment comparisons
Source: Res Synth Methods. 2026 Jul 3;17(6):1176–209. doi: 10.1017/rsm.2026.10106 (PMC13732385; doi:10.1017/rsm.2026.10106)
Supplement: Supplementary file 1 [file S1759287926101069sup001.pdf]

SUPPLEMENTARY MATERIAL

Targeting the average treatment effect in the treated

We briefly adapt the methodologies in Section 3.2 to Section 3.6 of the main text so that these target the ATT. We assume that there is full IPD availability and that $\hat{\mu}_1^1 = \frac{1}{n_1}\sum_{i=1}^{n_1} Y_i$ is consistent for μ_1^1.

For the modeling-based IOW approaches in Section 3.2, external control subjects are weighted by their conditional odds of SAT participation to transport the external control outcomes to the SAT (sub) population. SAT subjects are unweighted and external control subjects $i = n_1 + 1, \dots, n$ are weighted by $\hat{\omega}_i = \hat{e}_i/(1 - \hat{e}_i)$. Assuming correct specification of the propensity score model, the estimated weights would balance the covariate distribution of the external control with respect to that of the SAT, enabling consistent estimation of mean absolute outcome μ_1^0 and the ATT. Propensity score predictions that are close to one lead to extreme weights and imprecise ATT estimation, particularly where the sample size of the external control is small.

A MAIC estimator for the ATT, akin to that described in Section 3.3, would enforce that the covariate distributional features of the weighted external control subjects are exactly balanced with respect to those of the SAT subjects. As such, the balancing constraints would center the external control covariate balance functions on their SAT means. MAIC enables consistent estimation of μ_1^0 and the ATT, as long as either the log-odds of the propensity score or the potential outcome under the control are linear across the specified balance functions. The general form of the weighting estimators for the ATT is:

$$\widehat{\text{ATT}} = \underbrace{g\left(\frac{1}{n_1}\sum_{i=1}^{n_1} Y_i\right)}_{\hat{\mu}_1^1} - \underbrace{g\left(\frac{1}{K}\sum_{i=n_1+1}^{n} \hat{v}_i Y_i\right)}_{\hat{\mu}_1^0},$$

where K is a constant and $\hat{v}_i$ is a weight estimate for $i = n + 1, \dots, n$, derived using the modeling approach or MAIC.

A G-computation estimator such like that described in Section 3.4 but for the ATT requires postulating a model for the potential outcome expectation under the control, fitted to the external control participants. Based on the fitted model $m(\mathbf{X}_i; \hat{\boldsymbol{\beta}})$, the potential outcome under the control is predicted for each subject $i = 1, \dots, n_1$ in the SAT: $\hat{Y}_i^0 = q^{-1}\left(m(\mathbf{X}_i; \hat{\boldsymbol{\beta}})\right)$. The potential outcome predictions are averaged over the empirical covariate distribution of the SAT, resulting in the ATT estimator:

$$\widehat{\text{ATT}} = \underbrace{g\left(\frac{1}{n_1}\sum_{i=1}^{n_1} Y_i\right)}_{\hat{\mu}_1^1} - \underbrace{g\left(\frac{1}{n_1}\sum_{i=1}^{n_1} \hat{Y}_i^0\right)}_{\hat{\mu}_1^0}, \tag{36}$$

which relies on correct specification of the model for the potential outcome under the control for consistent estimation.

The DR augmented weighting estimators, proposed in Section 3.5, would target the ATT as follows. Based on an outcome model $m(\mathbf{X}_i; \hat{\boldsymbol{\beta}})$ fitted to the external control participants, the potential outcome under the control is predicted for all subjects $i = 1, \dots, n$ in the SAT and the external control: $\hat{Y}_i^0 = q^{-1}\left(m(\mathbf{X}_i; \hat{\boldsymbol{\beta}})\right)$. The potential outcome predictions are augmented with a weighted average of residuals for the external control subjects. The general form of the doubly robust augmented weighting estimators for the ATT is:

$$\widehat{\text{ATT}} = \underbrace{g\left(\frac{1}{n_1}\sum_{i=1}^{n_1} Y_i\right)}_{\hat{\mu}_1^1} - \underbrace{g\left(\frac{1}{K}\sum_{i=n_1+1}^{n} \hat{u}_i \epsilon_i^0 + \frac{1}{n_1}\sum_{i=1}^{n_1} \hat{Y}_i^0\right)}_{\hat{\mu}_1^0},$$

where K is a constant, $\hat{u}_i$ is a weight estimate and $\epsilon_i^0 = Y_i - \hat{Y}_i^0$ is a residual term for subject $i = n_1 + 1, \dots, n$ in the external control.

A weighted G-computation estimator akin to that described in Section 3.6 would target the ATT by: (1) estimating weights for the odds of SAT participation; (2) fitting a weighted model $m(\mathbf{X}_i; \hat{\boldsymbol{\beta}}_v)$ for the conditional outcome expectation to the external

control participants; and (3) averaging the outcome predictions of the weighted regression over the SAT covariate distribution. The resulting estimator for the mean absolute outcome μ_1^0 is $\hat{\mu}_1^0 = \frac{1}{n_1}\sum_{i=1}^{n_1}\hat{Y}_i^0 = \frac{1}{n_1}\sum_{i=1}^{n_1} q^{-1}\left(m(\mathbf{X}_i;\hat{\boldsymbol{\beta}}_v)\right)$, which is then substituted into Equation 36 for estimation of the ATT.

Plots of covariate overlap for the simulation study

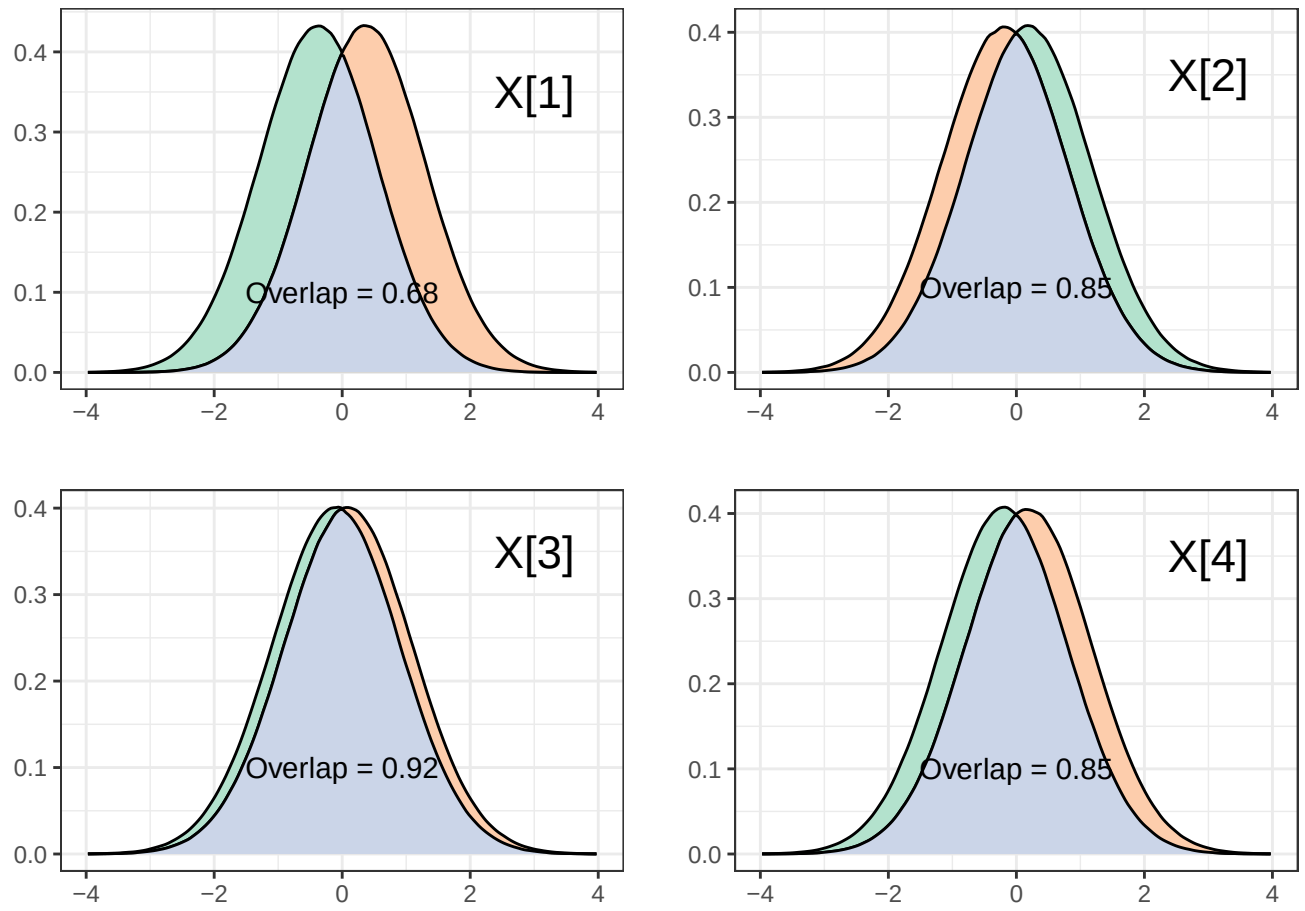

FIGURE 3 Density plots showing the overlap of covariates X_1,X_2,X_3, and X_4 for Scenarios KS1 and KS2 in the simulation study.

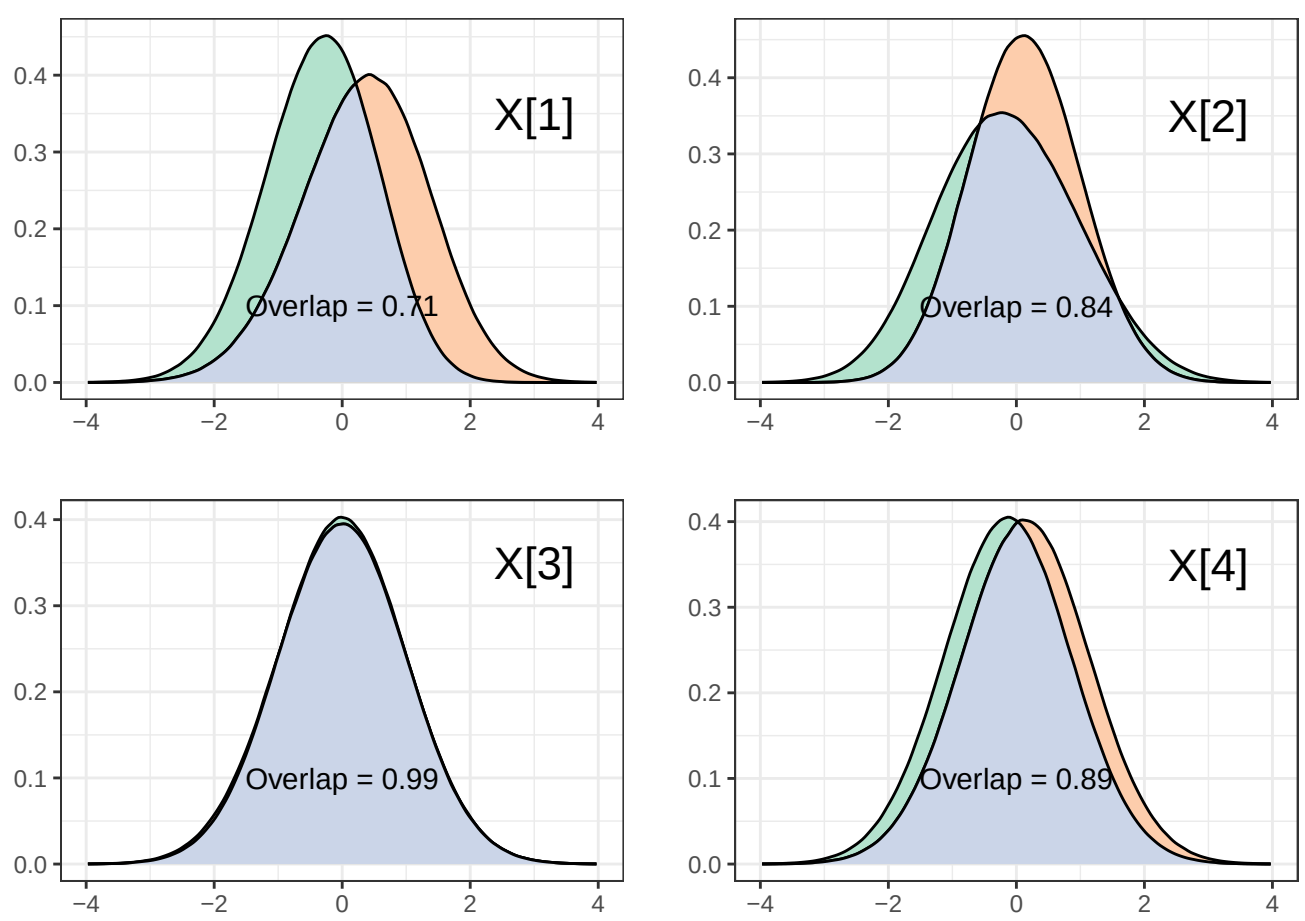

FIGURE 4 Density plots showing the overlap of covariates X_1,X_2,X_3, and X_4 for Scenarios KS3 and KS4 in the simulation study.
